# Effects of Sacubitril/Valsartan on biomarkers of fibrosis and inflammation in patients with heart failure with reduced ejection fraction

**DOI:** 10.1186/s12872-022-02647-0

**Published:** 2022-05-13

**Authors:** Giovanni Battista Bolla, Antonella Fedele, Andrea Faggiano, Carla Sala, Gloria Santangelo, Stefano Carugo

**Affiliations:** 1 Department of Clinical Sciences and Community Health, University of Milan and Fondazione IRCCS Ca’ GrandaOspedale Maggiore Policlinico, Milan, Italy; 2 Cardiology Unit, Internal Medicine Department, Fondazione IRCCS Ca’ Granda Ospedale Maggiore Policlinico, Milan, Italy; 3 Division of Cardiology, Department of Health Sciences, San Paolo Hospital, University of Milan, Milan, Italy

**Keywords:** Heart failure, Sacubitril/Valsartan, Biomarkers, Fibrosis, Left ventricular ejection fraction

## Abstract

**Aims:**

To evaluate the circulating levels of remodeling biomarkers procollagen type 1 C-terminal propeptide (PICP), human cartilage glycoprotein-39 (YKL-40), plasma renin activity (PRA), aldosterone (Aldo) as well as clinical and echocardiographic parameters in patients with heart failure with reduced ejection fraction (HFrEF), before and after treatment with Sacubitril/Valsartan (S/V).

**Methods and results:**

A total of 26 consecutive patients with HFrEF on stable clinical conditions were studied. Clinical, echocardiographic parameters and circulating biomarkers were measured at baseline, after 30 and 60 days of S/V treatment. Both systolic blood pressure (SBP) and diastolic blood pressure (DBP) decreased, from 126 ± 15 to 113 ± 4 mmHg (*p* < 0.001) and from 77 ± 11 to 72 ± 9 mmHg (*p* = 0.005), respectively, at the end of study. Concomitantly, left ventricular ejection fraction (LVEF) increased by 22.8% from 29.5 ± 5% to 36.2 ± 5%, (*p* < 0.001) and indexed left ventricular end-systolic volume (LVESVi) decreased by 12% from 38.6 ± 8.7 ml/m^2^ to 34.0 ± 10.0 ml/m^2^. (*p* = 0.007). Circulating levels of PICP, YKL-40, PRA and Aldo decreased by − 42.2%, − 46.8%, − 79.1% and − 76.7%, respectively (*p* < 0.001 for all), the decrements being already maximal within 30 days of S/V treatment. No significant changes of plasma electrolytes and creatinine were observed during the study (all *p* > 0.05).

**Conclusions:**

A decrease of circulating markers of inflammation and fibrosis during chronic treatment with S/V is associated with an improvement of hemodynamic and echographic parameters in patients with HRrEF. These data are compatible with an anti-fibrotic and anti-inflammatory effect of S/V, that may contribute to the beneficial outcomes of the drug in this clinical setting.

**Supplementary Information:**

The online version contains supplementary material available at 10.1186/s12872-022-02647-0.

## Introduction

Heart failure (HF) is a clinical syndrome characterized by ventricular remodeling and myocardial dysfunction leading to pump failure, increased morbidity and mortality, including sudden death [1, 2]. Left ventricle (LV) remodeling is characterized by diffuse reactive fibrosis resulting in increased ventricular stiffness, impaired diastolic and systolic function, reduced coronary flow reserve, and arrhythmias [3]. Fibrosis is a dynamic process characterized by excess collagen types I and III generation over degradation, leading to increased extracellular collagen deposition. During extracellular conversion of procollagen type I into mature collagen type I, type 1 procollagen C-terminal pro-peptide (PICP) is generated and released from the heart into peripheral circulation via the coronary sinus [4].

Moreover, circulating human cartilage glycoprotein-39 (YKL-40), a humoral marker associated with inflammatory conditions [5], has been reported to be significantly higher in patients with chronic HF compared to controls; this increment is apparently independent of drug treatment, including beta receptor blockers, angiotensin-converting-enzyme (ACE) inhibitors, angiotensin II receptor Antagonists (ARBs) and mineralocorticoid receptor antagonist (MRAs) [6]. High serum levels of YKL-40 may predict adverse cardiovascular events [7] and all-cause mortality in patients with chronic HF [8]. Activation of the renin-angiotensin- aldosterone system (RAAS) in HF contributes to adverse cardiac and vascular remodeling/fibrosis via angiotensin II (Ang II) type 1 receptors activation (AT1)[9]. Randomized controlled trials have demonstrated that RAAS blockade improves morbidity and mortality in HF patients with Reduced Ejection Fraction (HFrEF) [10, 11]. Sacubitril/Valsartan (S/V) is a novel drug combination designed to block the adverse effects of RAAS, to reduce bradykinin potentiation (which causes angioedema) by Valsartan, and to inhibit neprilysin inactivating effect of natriuretic peptides by Sacubitril metabolite LBQ657 [12, 13], thus leading to a natriuretic, vasodilatatory, and anti-proliferative effects. S/V has been proven to be superior to conventional ACE inhibition in reducing cardiovascular deaths (including sudden death) and HF readmissions in a large prospective randomized clinical trial [14].

The aim of this study was to evaluate the circulating levels of PICP, YKL-40, renin activity (PRA), aldosterone (Aldo), as well as clinical and echocardiographic changes induced by S/V treatment for 60 days in patients with HFrEF.

## Materials and methods

### Patient population

A prospective pharmacological, non-profit, monocentric interventional pilot study was conducted. After informed written consent for anonymous collection and publication of data was obtained, we prospectively enrolled 29 consecutive patients with HFrEF in stable hemodynamic conditions, eligible for S/V treatment according to the Guidelines [15]. All patients were followed at the Heart Failure Unit of Fondazione Cà Granda Ospedale Maggiore Policlinico, Milan, between 2019 and 2020.

All patients with HFrEF were on optimal medical therapy (OMT) before S/V, in particular 100% received beta receptor blockers, ACEI inhibitors or ARBs and MRAs. Hemodynamic stability was defined when systolic blood pressure (SBP) was at least 100 mm Hg, no increase of intravenous diuretic dose, no intravenous vasodilator injections had been required in the previous 6h neither intravenous inotropes in the previous 24 h. Patients with age less than 18 years, pregnancy, cardiac transplantation, ventricular assist device implantation and complex congenital heart disease were excluded. All study procedures had been approved by the Partners Healthcare Institutional Review Board (Practice # 601) and carried out in accordance with the Declaration of Helsinki.

### Data

Clinical data including age, sex, diastolic and systolic blood pressure (DBP-SBP), New York Heart Association (NYHA) class and therapy were obtained by reviewing each patient’s medical records. Clinical comorbidities were documented by review of medical charts, included hypertension (clinic blood pressure ≥ 140/90 mmHg or antihypertensive treatment), diabetes and dyslipidemia. Smokers included both current and former smokers. Ischemic etiology of HF was defined as history of myocardial infarction or prior coronary revascularization or stenosis ≥ 70% in ≥ 1 epicardial artery at coronary angiography.

Comprehensive transthoracic echocardiographic studies were performed by a single investigator (AF) at baseline and after 60 days of S/V treatment using a Philips Epiq 7. Briefly, LV end-diastolic volume (LVEDV) and LV end-systolic volume (LVESV) were measured from the apical two- and four-chamber views using the biplane modified Simpson’s rule, LV ejection fraction (LVEF) was calculated as (LVEDV-LVESV)/LVEDV × 100 [16].

Clinical and laboratory data were collected at baseline, after 30 and 60 days of S/V treatment. Blood samples were collected in the morning, with the patient in the seated position, centrifuged and stored at 80°C until analysis. Serum YKL-40 and PICP were measured by ELISA with commercially available kits (MicroVue Bone for YKL 40, normal values 25–125 ng/mL; EIA, Quidel Company for PICP, normal values 69–163 ng/mL). Radio-Immunossay kits (Beckman Coulter Company) were used to measure plasma renin activity (PRA; normal values 0.167−5.380 ng/mL/h) and plasma aldosterone (Aldo; normal values 4.0–31.0 ng/dL, N-terminal pro B-type natriuretic peptide (NT-proBNP) was measured by immuno-chemo-luminescence method (Cobas Roche).

### Statistical analysis

All observed patient data was included in the Microsoft Excel package. All the hypotheses under study were verified through the use of the statistical packages SPSS and Statistics (StatSoft). The significance level (α) of the statistical tests used for the evaluation were considered at a two-tailed probability level of significance of 95% (*p* < 0.05). The choice of tests (parametric and non-parametric) was made after verifying the normality distribution of the data with the Kolmogorov Smirnov test. The variables observed over time T0-T30-T60 were verified by using the method of analysis of variance for repeated measures (Anova way). The presence of a statistically significant difference between the times was confirmed with the Tukey multiple comparison method. While for the variables observed at time T0-T60 the T tests (Student's) for paired data was used.

## Results

A total of 29 consecutive patients were enrolled in the study; as 3 patients died during the study period, one due to extra-cardiac causes, the final analysis included 26 patients**.** Their age was 69.8 ± 2.3 years, 20 (77%) were men, 12 patients had ischemic heart disease, all patients were in NYHA class III; an isolated Cardioverter-Defibrillator (ICD) had been implanted in 6 pts and a Cardiac Resynchronization Therapy-Defibrillator (CRT-D) in 8 pts at least 6 months before study. Table 1 shows baseline characteristics of the study population.

**Table 1:** Baseline characteristics of the study population

Patients, N = 26	
Age, years	69.8 ± 2.3
Men, n (%)	20 (77%)
Ischemic etiology, n (%)	12 (46%)
NYHA class III, n (%)	26 (100%)
SBP (mmHg)	126.8 ± 3.1
DBP (mmHg)	77.5 ± 2.1
Creatinine (mg/dl)	1.1 ± 0.1
eGFR (ml/min/1.73m^2^)	44.3 ± 4.1
Na^+^ (mEq/L)	139.9 ± 0.5
K^+^ (mEq/L)	4.4 ± 0.1
Smoking history, n (%)	13 (50%)
Diabetics, n (%)	10 (38.4%)
Hypertension, n (%)	16 (62%)
Hyperlipidemia, n (%)	16 (62%)
BB, n (%)	26 (100%)
MRA, n (%)	26 (100%)
Diuretics, n (%)	26 (100%)
Sinus Rythm, n (%)	17 (65%)
Atrial Fibrilation, n (%)	4 (15%)
ICD, n (%)	6 (23%)
CRT-D, n (%)	8 (31%)
LNEF (%)	29.5 ± 1.0
LVESVi (ml/m^2^)	38.6 ± 8.7

*NYHA* New York Heart Association; *SBP* systolic blood pressure; *DBP* diastolic body pressure; *eGFR* estimated glomerular filtration rate calculated with Chronic Kidney Disease Epidemiology Collaboration (CKD-EPI) formula; *Na+ * sodium; *K+* potassium; *BB* beta-blockers; *ICD* implantable cardioverter defibrillator; *MRA* mineralcorticoid receptor antagonist; *CRT-D* cardiac resynchronization therapy—defibrillator; *LVEF* Left ventricular ejection fraction; *LVESVi* Indexed Left ventricular end-systolic volume. Values are presented as absolute numbers, percentages and mean ± standard error (SE)

All patients were on OMT before S/V treatment; they were switched from an ACEi or ARBs to S/V, initially prescribed at 24/26 mg twice a day [17]: this dose was maintained in 16 pts (61,5%), S/V was up titrated to maximum tolerated dose (49/51 mg twice a day) in 5 patients (19,2%) or target dose (97/103 mg twice a day) in 5 patients (19,2%) within 30 days of treatment. No patients were hospitalized and none developed decompensated symptoms. All patients are still treated. No adverse events occurred during therapy.

### Effect of S/V treatment

SBP decreased from 126 ± 3.0 at baseline to 115±2.9 mmHg after 30 days of S/V (*p*<0.001) and levelled-off to 113.4 ± 3.2 mmHg after 60 days with an average reduction of − 10.0%. Similarly, DBP decreased from 77.5 ± 2.1 mmHg at baseline to 71.9 ± 1.7 mmHg after 30 days (*p* = 0.005) and levelled-off to 72.8 ± 1.7 mmHg after 60 days with an average reduction of − 6.0%; no episodes of acute hypotension were recorded. No changes of creatinine and electrolytes values were observed throughout study (all *p* > 0.05).

**Fig. 1:**
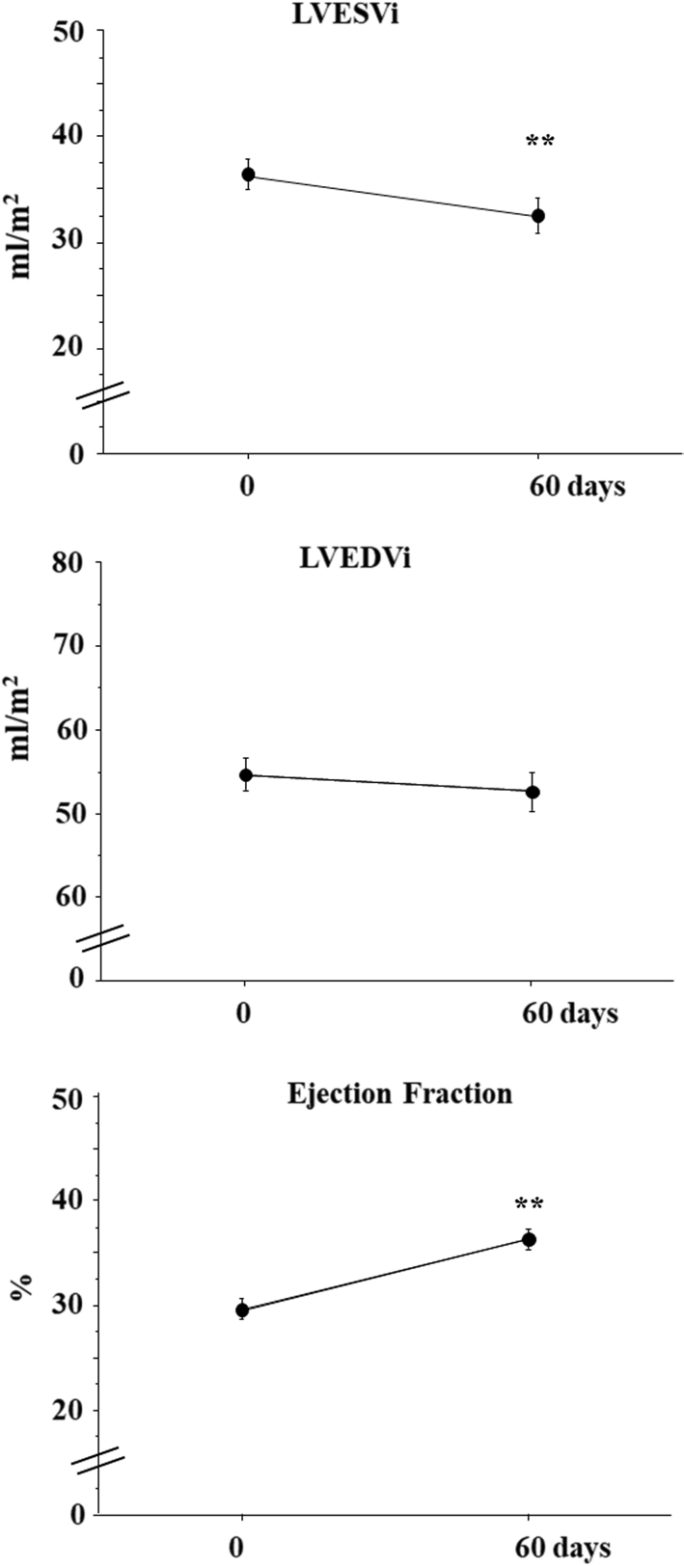
Echocardiographic data at baseline and after 60 days of S/V treatment. LVESVi: Indexed left ventricular end-systolic volume. LVEDVi: Indexed left ventricular end-diastolic volume. LVEF: Left ventricular ejection fraction. Means ± se; **p < 0.01

Compared to baseline, indexed left ventricular end-systolic volume (LVESVi) decreased from 38.6 ± 8.7 ml/m^2^ to 34.0 ± 10.0 ml/m^2^ after 60 days of S/V treatment (*p* = 0.007), with an average reduction of − 12.0%; no significant changes of indexed left ventricular end-diastolic volume (LVEDVi) were observed (from 55.9 ± 11.8 ml/m^2^ to 53.4 ± 13.8 ml/m^2^, *p* = 0.12). As a result, LVEF increased from 29.5 ± 1.0% to 36.2 ± 1.0%, (*p* < 0.001), with an average change of +22.8% (Fig. 1).

**Fig. 2:**
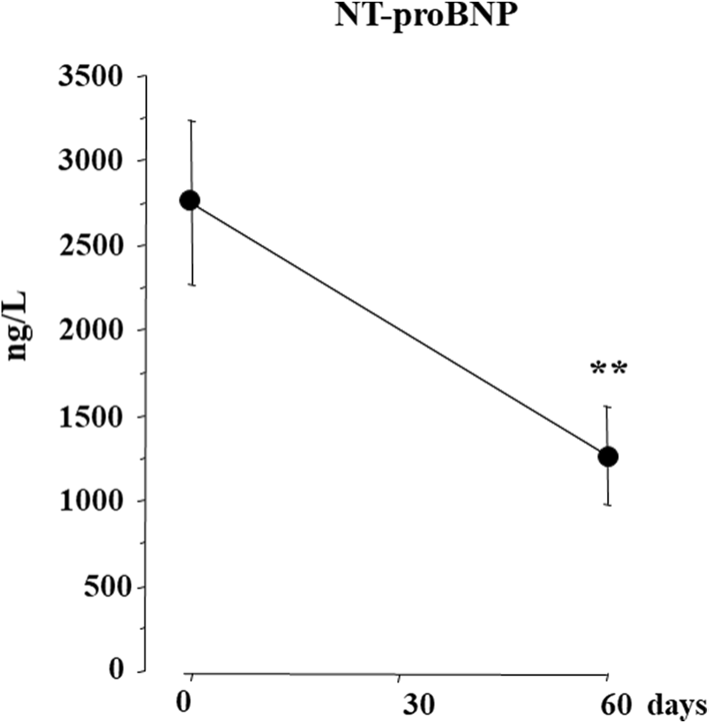
Plasma levels of N-Terminal pro B-type natriuretic peptide (NT-proBNP) at baseline and after 60 days of S/V treatment. Means ± se; **p < 0.01

Compared to baseline, NT-proBNP decreased from 2769 ± 481 ng/L to 1282 ± 289 ng/L after 60 days of S/V, with a percent decrement of − 53.7% (Fig. 2).

**Fig. 3:**
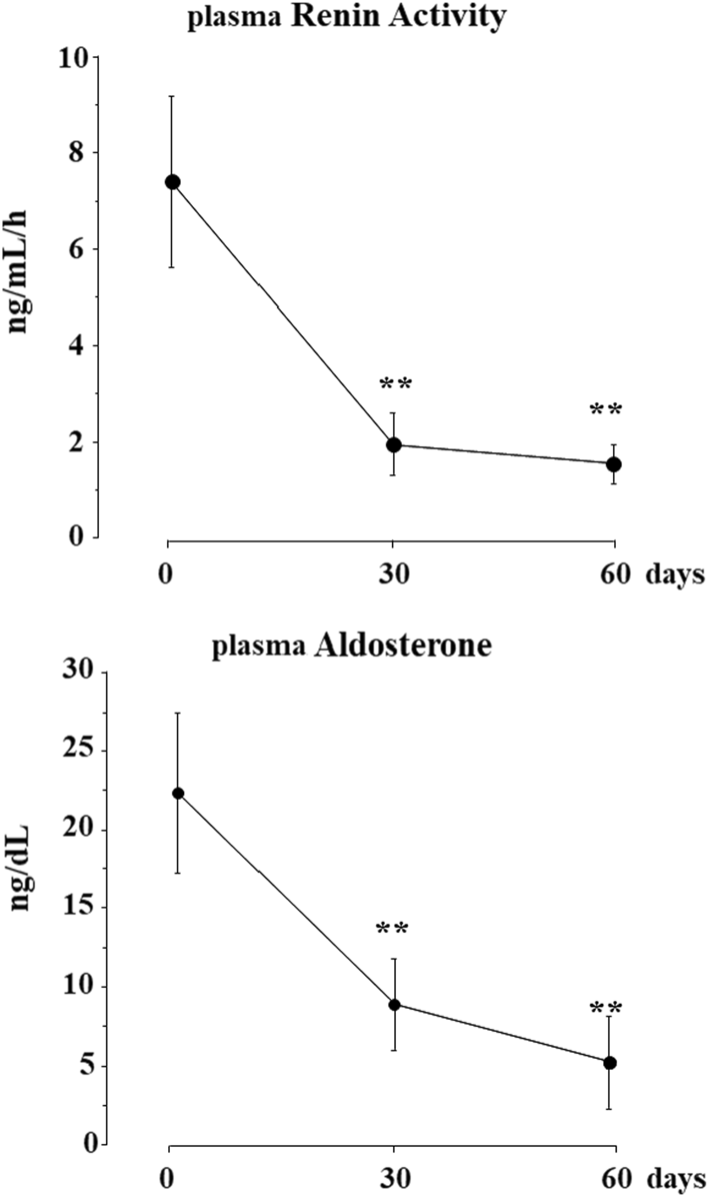
Plasma levels of renin activity and aldosterone at baseline and after 30 and 60 days of S/V treatment. Means ± se; **p < 0.01 versus baseline

Baseline PRA was 7.47 ± 1.78 ng/mL/h and Aldo was 22.3 ± 5.12 ng/dL; after 30 days of S/V, PRA was 1.97 ± 0.64 ng/ml/h and Aldo was 8.87 ± 2.9 ng/dl, with a percent reduction of − 73.6% and − 60.9%, respectively (*p* < 0.001 for both). These decrements persisted after 60 days of S/V therapy: PRA was 1.55 ± 0.40 ng/ml and Aldo 5.19 ± 3.4 ng/dl, percent decrements from baseline being − 79.2% and − 76.7%, respectively (*p* < 0.001 for both) (Fig. 3).

### Fibrosis and inflammation biomarkers

**Fig. 4:**
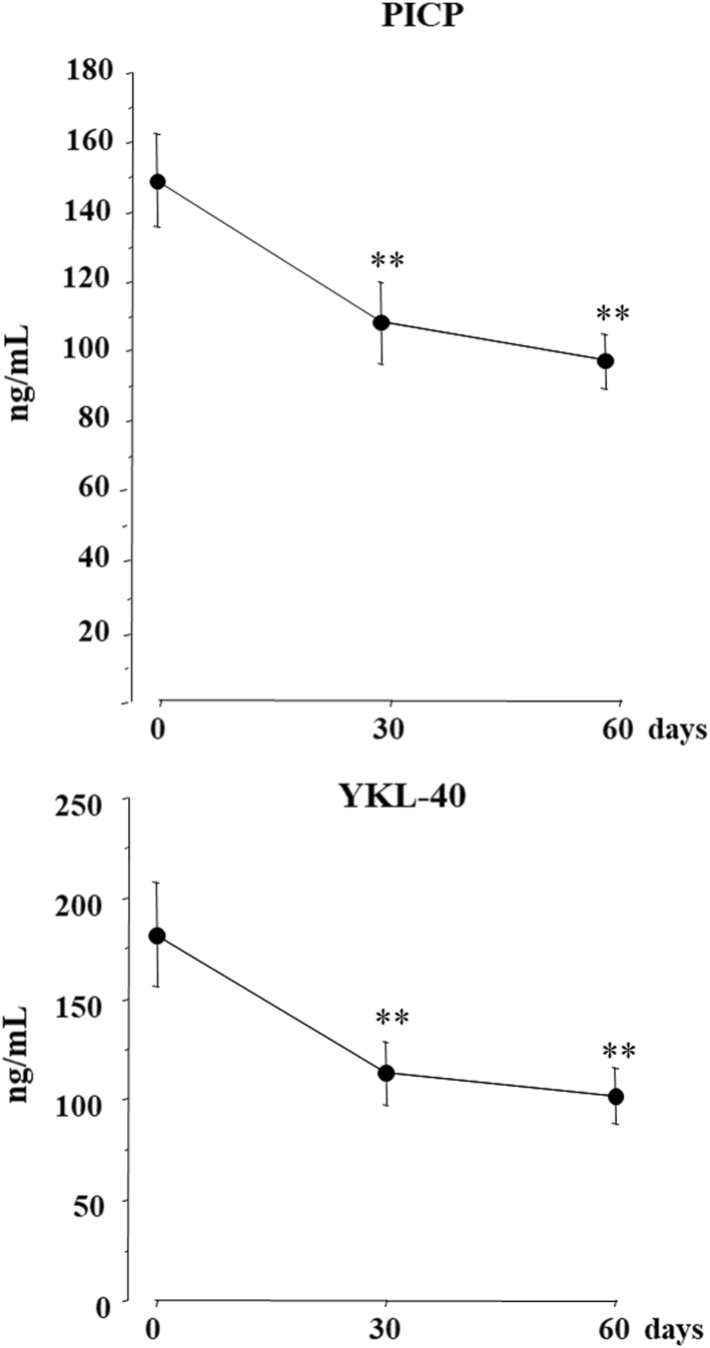
Serum PICP: Type 1 procollagen C-terminal propeptide (PICP) and Human cartilage glycoprotein-39 (YKL-40) at baseline and after 30 and 60 days of S/V treatment. Means ± se; **p < 0.01 versus baseline

At baseline, PICP was 147.8 ± 16.0 ng/ml and YKL-40 was 191.8 ± 25.8 ng/ml; after 30 days of S/V, PICP was 98.5 ± 19.3 ng/ml and YKL-40 was 113.2 ± 15.6 ng/ml, with a percent decrease of − 33.4% and − 40.9%, respectively (*p* < 0.001 for both). After 60 days, PICP was 85.4 ± 16.8 ng/ml and YKL-40 was 101.9 ± 13.8 ng/ml, the percent reduction from baseline being − 42.2% and − 46.8% (*p *< 0.001 for both) (Fig. 4). The single patients values of PICP and YKA-40 throughout the study has been provided in the Additional file 1: Figs. S1 and S2. To understand whether the S/V dose could have an influence on the remodeling and inflammation biomarkers, non-parametric tests were carried out. Precisely no statistically significant difference between the three dosages (24/26 mg, 49/51 mg, 97/103 mg) was found for either of the variables (PICP and YKL-40) at the three times of detection: T0 (*P* value for PICP = 0.77, *P* value for YKL-40 = 0.35), T1 (*P* value for PICP = 0.96, *P* value for YKL-40 = 0.75), T2 (*P* value for PICP = 0.88, *P* value for YKL-40 = 0.32). Instead, as expected, we found significant changes in PICP and YKL-40 values within times for all the three S/V doses. All the percentual variations within times are expressed in the attached Additional file 1: Tables S1 and S2.

## Discussion

The major finding of our study was that S/V treatment in patients with HFrEF was associated with a decrease of circulating markers of fibrosis and inflammation. This effect was already present within 30 days of treatment, persisted during the following 30 days and was associated with a progressive inhibition of the RAAS. The hemodynamic effect of S/V, a decrease of systolic/diastolic BP by 14/4 mmHg, was associated with improved echocardiographic parameters of reverse remodeling and left ventricular systolic function, as documented by the reduction in LVESVi and the increase in LVEF. The concomitant 53% decrease of plasma NT-proBNP after S/V treatment is compatible with reduced cardiac filling pressures and myocardial strain. The circulating levels of this peptide have been shown to accurately predict cardiovascular outcomes in HFrEF [18]. Of note, NT-proBNP, unlike Atrial Natriuretic Peptide (ANP) and Brain Natriuretic Peptide (BNP), is not a substrate for neprylisin and therefore its circulating levels properly reflect the cardioprotective effects of S/V. The enhanced biological activity of the natriuretic peptide system after blockade of neprylisin has been documented by the increased plasma levels of ANP, BNP and their second messenger cyclic guanosine monophosphate (cGMP) [12]. As both these natriuretic peptides are known to inhibit renin and aldosterone secretion, it is conceivable that their enhanced activity was responsible for the decrease of PRA and aldosterone observed in our study. The elevated baseline levels of PRA, indeed, were 75% lower after 30 days of S/V treatment and the decrement persisted until the end of the study. Similarly, aldosterone decreased by 60% and 76% compared to baseline after 30 and 60 days of treatment, respectively.

Myocardial fibrosis is a pathophysiological mechanism involved in the development and progression of chronic HF and the hallmark feature of adverse LV remodeling [19]. The extent and distribution of myocardial fibrosis is the final result of homeostatic processes regulating cardiac extracellular matrix (ECM) composition. Cardiac ECM is mostly represented by collagen type I (85%) and III (11%) which are synthetized by cardiac fibroblasts and undergo complex processing until final degradation [20]. Collagen synthesis by fibroblasts is enhanced by the activated RAAS system in patients with HFrEF. In this context, PICP may be seen as a marker of collagen type I synthesis and elevated serum PICP levels have been reported in patients with HFrEF compared to controls [21]. The adverse ventricular fibrosis and remodeling induced by neurohormonal and hemodynamic alterations in HFrEF patients is reflected by morpho-structural cardiac changes highlighted by non-invasive measures of LVEF, LVESV and LVEDV. These structural changes are associated with an increased risk of atrial and ventricular arrhythmias and reduced myocardial perfusion. The presence and extent of fibrosis adversely affects morbidity and mortality in patients with HF [22]. Massoullie et al [2] showed that non-responder CRT patients had higher serum PICP levels, thus suggesting that evaluation of PICP prior to CRT implantation may contribute to identify patients prone to respond to CRT.

To our knowledge, this study is the first to evaluate the effects of S/V on myocardial fibrosis with serial monitoring of the fibrotic biomarker PICP [23, 24]. A recent study by Zile et al. documented that S/V decreased some biomarkers of profibrotic signaling (aldosterone, Soluble Isoform of ST2, Tissue Inhibitor of Metalloproteinase-1, Galectin-3 Protein, Procollagen Type I N-propeptide, and Type III Procollagen Peptide in HFrEF patients, thus suggesting that S/V may reduce myocardial fibrosis and improve clinical outcomes. The authors also found that some of these profibrotic biomarkers reflecting determinants of ECM homeostasis had a significant prognostic value [25]. Our study, unlike the Zile's study, explored a serum biomarker of fibrosis (PICP) which best correlates with the extent of fibrotic deposition in cardiac tissue as demonstrated by endomyocardial biopsies [26].

Of note, this effect of S/V was observed in patients chronically treated with inhibitors of the renin-angiotensin aldosterone system, including MRAs, which have been documented to directly affect fibrosis markers. Whether these decrements may be related to the enhanced activity of the natriuretic system by S/V or to a more potent inhibition of the RAAS remains to be determined.

Our finding that S/V provides potential antifibrotic effects may contribute to better understand its mechanisms of action. Moreover, defining the fibrotic profile of HFrEF patients eligible to S/V treatment, may improve the selection criteria and maximize the beneficial effects of this compound [27].

Systemic inflammation in patients with HF is the result of tissue hypoperfusion and neurohormonal activation [19]. The immune system plays a significant role in HF progression, ventricular remodeling, and long-term cardiac injury. In particular, activation of a variety of inflammatory pathways, such as the complement system, T cells, and synthesis of autoantibodies, have been reported in HF patients. Overall, the inflammatory process contributes to the worsening of HF syndrome, by inducing cachexia and anemia [18]. The inflammatory marker high-sensitivity C-reactive protein is elevated in HF patients, its circulating levels further increase during episodes of clinical decompensation and predict worse cardiac outcomes [28]. Of note, in a recent case series analysis S/V significantly reduced the circulating levels of high-sensitivity C-reactive protein within 4 weeks of treatment in patients with acute decompensated HF [18]. YKL-40 is a highly conserved heparin-, chitin-, and collagen-binding glycoprotein, mainly produced by macrophages, neutrophils and cancer cells in inflammatory states. YKL-40 regulates vascular endothelial growth factor and has a role in the processes of inflammation, angiogenesis, cell proliferation and differentiation, as well as ECM remodeling [9]. In a recent study in HF patients, serum YKL-40 was significantly associated with all-cause mortality, after multivariable adjustment for cardiovascular risk factors [9].

Our study for the first time documented that S/V therapy was able to reduce the inflammatory context of HF, as demonstrated by the rapid decrease of YKL-40 values (− 46.8% compared to baseline), that was already evident within 30 days of treatment and persisted until the end of study.

### Limitations

A control group was not included in the protocol for ethical reasons; a propensity-score match control study, however, may partially address this limitation.

Markers of fibrosis were measured in the systemic circulation, thus the origin of these compounds from other sources than cardiac tissue is not excluded, as cardiac biopsies were not performed in this study. It should be underlined, however, that serum PICP concentrations have been shown to be directly related to the fibrillar collagen fraction of the myocardium as documented by endomyocardial biopsies [26]. Future studies with cardiac magnetic resonance imaging, the gold standard non-invasive technique for detecting fibrosis, are highly desirable in order to support this hypothesis.

The limited sample size of the study is related to difficulties in enrolling a larger number of patients due to the concomitant Covid-19 pandemic. Anyway, this is an exploratory study that succeeded in demonstrating a substantial modification of biohumoral values and biomarkers despite the small sample size and the low percentage of patients receiving S/V target dose, suggesting that these data can be easily replicated and reinforced by larger studies. The follow-up period is limited but the finding that positive results have come so early from the introduction of S/V therapy supports the hypothesis that these benefits could be maintained and prove relevant in the long term. A longer follow-up is needed to confirm this hypothesis.

## Conclusions

The study documented a decrease of circulating markers of fibrosis and inflammation, PICP and YKL-40, in HFrEF patients after S/V administration, thus supporting a reduced inflammatory/profibrotic state associated with this treatment. These effects were already maximal within 30 days of treatment, persisted for the following 30 days and were likely related to the enhanced activity of natriuretic peptide system and inhibition of RAAS induced by S/V. Future studies in larger samples are highly desirable in order to clarify the long-term effect of S/V on fibrosis in cardiac tissue.

## Supplementary Information

**Additional file 1: Figure S1.** Single patients values of PICP throughout the study. PICP = Type 1 procollagen C-terminal propeptide. **Figure S2.** Single patients values of YKL-40 throughout the study. YKL-40 = Human cartilage glycoprotein-39. **Table S1.** Percentual variations of PICP values within times according to the different S/V dosage. S/V = Sacubitril/ Valsartan. PICP = Type 1 procollagen C-terminal propeptide. **Table S2.** Percentual variations of YKL-40 values within times according to the different S/V dosage. S/V = Sacubitril/ Valsartan, YKL-40 = Human cartilage glycoprotein-39.

## Data Availability

The datasets generated and/or analysed during the current study are not publicly available due to investigators’ choice but are available from the corresponding author on reasonable request**.**
