# Supplementary material for: Effects of Sacubitril/Valsartan on biomarkers of fibrosis and inflammation in patients with heart failure with reduced ejection fraction
Source: BMC Cardiovasc Disord. 2022 May 13;22:217. doi: 10.1186/s12872-022-02647-0 (PMC9101988; doi:10.1186/s12872-022-02647-0)
Supplement: Supplementary file 1 — Additional file 1: Figure S1. Single patients values of PICP throughout the study. PICP = Type 1 procollagen C-terminal propeptide. Figure S2. Single patients values of YKL-40 throughout the study. YKL-40 = Human cartilage glycoprotein-39. Table S1. Percentual variations of PICP values within times according to the different S/V dosage. S/V = Sacubitril/ Valsartan. PICP = Type 1 procollagen C-terminal propeptide. Table S2. Percentual variations of YKL-40 values within times according to the different S/V dosage. S/V = Sacubitril/ Valsartan, YKL-40 = Human cartilage glycoprotein-39. [file 12872_2022_2647_MOESM1_ESM.docx]

Additional file 1


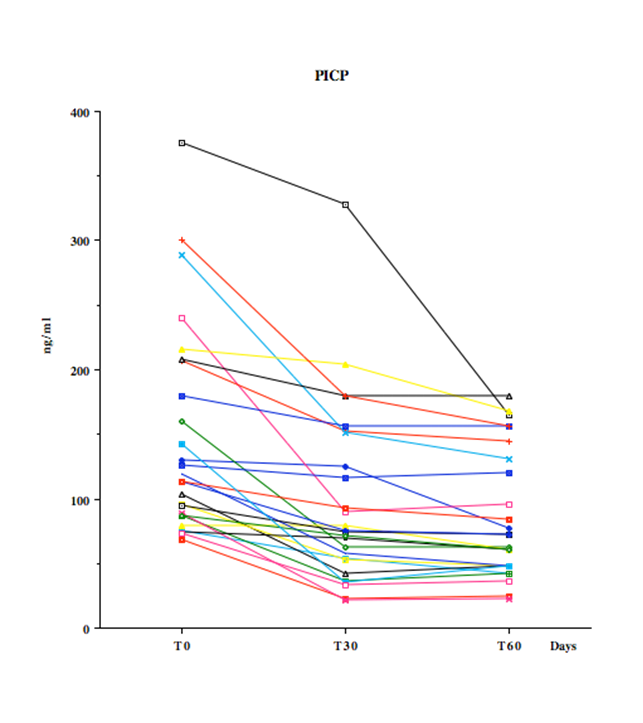


Figure S1: Single patients values of PICP throughout the study. PICP = Type 1 procollagen C-terminal propeptide.


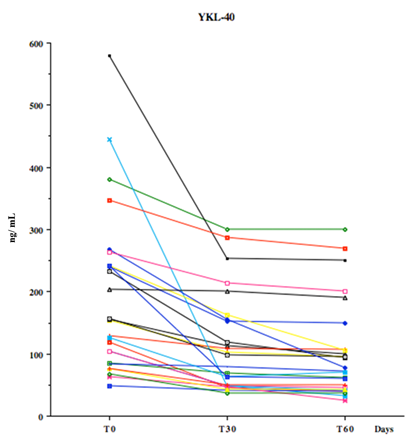


Figure S2: Single patients values of YKL-40 throughout the study. YKL-40 = Human cartilage glycoprotein-39

Table S1: Percentual variations of PICP values within times according to the different S/V dosage. S/V = Sacubitril/ Valsartan. PICP = Type 1 procollagen C-terminal propeptide

| **Table S1** | | **PICP values % variations versus T0** | | | |
| --- | --- | --- | --- | --- | --- |
| Patients | S/V Dose | T1 (30 Days) | P value vs T0 | T2 ( 60 Days) | P value vs T0 |
| N = 16 | Dose 24/26 mg/dl | -31.6 % | 0.0007 | -35.7 % | 0.0004 |
| N = 5 | Dose 49/51 mg/dl | -24.6% | 0.04 | -51.2% | 0.04 |
| N = 5 | Dose 97/103 mg/dl | -46.1 % | 0.04 | -51.1% | 0.04 |

Table S2: Percentual variations of YKL-40 values within times according to the different S/V dosage. S/V = Sacubitril/ Valsartan, YKL-40 = Human cartilage glycoprotein-39

| **Table 2** | | **YKL-40 values % variations versus T0** | | | |
| --- | --- | --- | --- | --- | --- |
| Patients | S/V Dose | T1 (30 Days) | P value vs T0 | T2 ( 60 days) | P value vs T0 |
| N = 16 | Dose 24/26 mg/dl | -41.9% | 0.0004 | -45.5% | 0.0004 |
| N = 5 | Dose 49/51 mg/dl | -37.6% | 0.04 | -45.2% | 0.04 |
| N = 5 | Dose 97/103 mg/dl | -39.8 % | 0.04 | -56.2% | 0.04 |
